# Nutraceutical Additives Modulate Microbiota and Gut Health in Post-Weaned Piglets

**DOI:** 10.3390/vetsci11080332

**Published:** 2024-07-25

**Authors:** Jaime A. Ángel-Isaza, Víctor Herrera Franco, Albeiro López-Herrera, Jaime E. Parra-Suescun

**Affiliations:** 1 Doctorado en Biotecnología, Facultad de Ciencias, Universidad Nacional de Colombia sede Medellín, Medellín 050034, Colombia; 2 Unidad de Investigación, Innovación y Desarrollo Promitec Santander, Bucaramanga 680002, Colombia; 3 Grupo de Investigación Biodiversidad y Genética Molecular (BIOGEM), Universidad Nacional de Colombia Sede Medellín, Medellín 050034, Colombia; vhherrer@unal.edu.co (V.H.F.); alherrera@unal.edu.co (A.L.-H.); jeparrasu@unal.edu.co (J.E.P.-S.); 4 Facultad de Medicina Veterinaria, Corporación Universitaria REMINGTON, Medellín 050034, Colombia; 5 Facultad de Ciencias Agrarias, Departamento de Producción Animal, Universidad Nacional de Colombia Sede Medellín, Medellín 050034, Colombia

**Keywords:** phytobiotic, nutraceutical, nutrition, prebiotic, swine, leaky gut

## Abstract

**Simple Summary:**

After piglet weaning, with the exposure to the inherent stress of this productive stage, the intestinal microbiota experiences an imbalance, resulting in a loss of beneficial microbial diversity and the development of potentially pathogenic or opportunistic bacteria. This leads to diarrhea and a decrease in the productivity and parameters of intestinal integrity and health. To address this issue, growth-promoting antibiotics have been used. However, in recent years, the search for nutraceutical compounds that can improve intestinal microbial balance and, thus, reduce antibiotic use has become important. In this regard, the effect of four nutraceutical compounds on piglet intestinal microbiota was evaluated for 30 days post-weaning. Following bioinformatic analyses, we report that essential oil from *Lippia origanoides* and short-chain fructo-oligosaccharides showed promising effects in reducing weaning-induced intestinal dysbiosis, with a decrease in potentially pathogenic taxa and an increase in bacteria associated with improved productivity and morphometric variables of the intestine, as well as the expression of intestinal barrier proteins. These findings elucidate the mechanisms of microbiota modulation by these compounds, aiming to reduce the use of prophylactic antibiotics and promoting the development of new studies focused on exploring metabolic changes in intestinal microbial communities.

**Abstract:**

Due to the challenge of weaning pigs and the need to reduce the use of antimicrobials in animal feed, there is a growing need to look for nutraceutical alternatives to reduce the adverse effects of the post-weaning period. We evaluate the effect of different feed nutraceutical additives on the microbial communities, gut health biomarkers, and productivity of pigs during the post-weaning period. The study involved 240 piglets weaned on the 21st day of age and randomized to six different diets: D1-BD commercial standard feed, D2-AGP: D1 + 150 ppm zinc bacitracin, D3-MD: D1 + 550 ppm maltodextrin, D4-FOS: D1 + 300 ppm fructo-oligosaccharides, D5-EO: D1 + 70 ppm *Lippia origanoides* essential oil, and D6-SH: D1 + 750 ppm sodium humate. On day 30 post-weaning, zootechnical parameters were evaluated, and jejunal samples were taken to obtain morphometric variables, expression of barrier and enzymatic proteins, and analysis of microbial communities. Animals fed D4-FOS and D5-EO had the lowest feed conversion ratio and higher expression of barrier and enzymatic proteins compared to D1-BD, D2-AGP, and D3-MD. The use of the additives modified the gut microbial communities of the piglets. In conclusion, fructo-oligosaccharides and *Lippia origanoides* essential oil were the best alternatives to zinc bacitracin as antibiotic growth promoters.

## 1. Introduction

The weaning process in swine production exposes piglets to several stressors, including changes in diet, environment, and social context. These stressors, in turn, are accompanied by bacterial challenges caused by imbalances in gut microbial communities [1]. This deterioration in the balance of the gut microbiota is associated with impaired gut integrity, digestive disorders, and health problems, leading to a reduction in piglet growth rate and the increased occurrence of diarrheal episodes [2]. During the first weeks after weaning, gut microbiota diversity declines, causing a reduction in the abundance of bacterial groups capable of producing short-chain fatty acids (SCFAs), metabolites known for their health-promoting benefits [3]. Additionally, this stage is characterized by an increase in pathobionts or opportunistic pathogens in the intestine, such as enterotoxigenic Escherichia coli [4].

An increase in pathobionts causes an imbalance in intestinal homeostasis that negatively affects the expression of intestinal proteins associated with brush border enzymes (disaccharidases and aminopeptidases) and tight junction barrier proteins (claudin—CL, occluding—OC, and zonula occludens—ZO) [1,5]. These proteins play a significant role in regulating permeability, which keeps intestinal pathogenic micro-organisms, endotoxins, and other antigenic substances from crossing the intestinal mucosal barrier, reaching the bloodstream, and spreading to other organs, resulting in enteroborne infections [6].

This phenomenon has traditionally been treated with prophylactic antibiotic growth promoters (AGPs). However, developing nutritional strategies to replace AGPs is necessary in light of new industry and market trends to reduce the use of antimicrobials in animal husbandry as part of the fight against antimicrobial resistance. In recent years, there has been an increase in research and development focused on natural alternatives, including prebiotics, acidifiers, and phytobiotics, such as essential oils, to prevent intestinal disorders in piglets during the post-weaning period [1,7]. 

Within this group of alternatives, short-chain fructo-oligosaccharides (FOS), such as kestose, nystose, and fructofuranosylnystose, are oligosaccharides composed of linear polymers of D-fructose linked by β-glycosidic bonds. Due to this structure, they are resistant to digestion by pig intestinal enzymes, making them selective substrates for fermentation by bacteria present in the intestinal microbiota [8,9]. Supplementation with these prebiotics stimulates microbial production of SCFAs and modulates the composition of the animals’ intestinal microbiota [10]. On the other hand, essential oils (EO) are complex mixtures of volatile compounds, predominantly low molecular weight terpenoids and phenylpropanoids [11]. Among them, the EO of *Lippia origanoides*, rich in thymol and carvacrol, is widely used in animal nutrition due to its bactericidal and antioxidant properties, which can modulate intestinal health and influence the microbial communities of animals [12,13,14].

Sodium humates (SH) are sodium salts of humic acid obtained by treating organic sources rich in humic substances such as lignite, coal, or weathered coal. These compounds possess a remarkable ion exchange capacity with heavy metal ions, as well as strong adsorption, complex formation, and chelation capabilities [15]. Consequently, they have been widely employed in various agro-industrial applications [16], including animal nutrition [17], where they have been the subject of research due to their potential to reduce the incidence of diarrhea during the post-weaning period [18].

These nutraceutical compounds, which have been proposed as alternatives to AGPs (FOS, MD, EO, and SH), have the ability to interact with the intestinal microbial communities of animals. However, the mechanism of interaction by which each of these compounds relates to intestinal micro-organisms differs [19,20,21,22]. Our hypothesis is that each of the mentioned nutraceutical compounds may exert a differentiated modulation of the ileal microbiota in piglets during the post-weaning period, thus influencing the small intestinal health of the animals in distinct ways.

Therefore, the present study aimed to evaluate the effect of different feed additives (*Lippia origanoides* essential oil, short-chain fructo-oligosaccharide, sodium humate, and maltodextrin) on the ileal microbial communities and gene expression (mRNA) of enzymatic and barrier proteins in the small intestine of pigs during the post-weaning period.

## 2. Materials and Methods

### 2.1. Ethical Considerations

The research was approved by the Institutional Committee for the Care and Use of Animals (CICUA, for its acronym in Spanish) at the headquarters of the Universidad Nacional de Colombia sede Medellín (CICUA-19-22, Minute 09, 8 November 2022). All animal experiments followed the CCAC Guidelines on the care and use of farm animals in research [23].

### 2.2. Experimental Setup and Animals

The fieldwork took place in a commercial pig farm nestled in the village of “El Zancudo,” municipality of Entrerríos (Antioquia, Colombia). The property is located at an elevation of approximately 2300 m (7546 feet) above sea level in a very humid low montane forest life zone (bmh-MB). The temperature ranges between 18 °C and 24 °C, with an average monthly rainfall of 208 mm and a relative humidity of 70%.

A total of 240 male piglets (PIC genetic line) weaned at 21 days of age with an average weight of 6.61 ± 0.08 kg were randomly selected on the day of weaning and housed in groups of eight in 5 m^2^ pens with a final stocking density of 0.35 m^2^ per animal, following an experimental design consisting of five replicates of six treatments, where each pen of eight animals constituted the experimental unit [24]. Animals had access ad libitum to water and food throughout the experimental period, and their food consumption was recorded using management practices and following commercial procedures. No solid feed or antibiotic compounds were fed to the piglets during lactation.

### 2.3. Experimental Diets

During the experimental period, animals were fed a balanced diet formulated using Microsoft^®^ Excel Solver for minimum cost optimization, following the NRC [25] recommendations. This basal diet (BD) was free of antibiotic growth promoters (AGPs) and feed additives (Table 1). For each of the treatments, an AGP or one of the four nutraceutical additives was added to the BD to create each experimental diet as follows: D1-BD: basal diet (BD): balanced feed without nutraceutical additives or antibiotics; D2-AGP: BD + 150 ppm zinc bacitracin; D3-MD: BD + 550 ppm maltodextrin (MD); D4-FOS: BD + 300 ppm short-chain fructo-oligosaccharides; D5-EO: BD + 70 ppm *Lippia origanoides* essential oil, and D6-SH: BD + 750 ppm sodium humate. All nutraceutical additives used in the study were supplied by Promitec^®^ Santander (Promitec Santander S.A.S., Bucaramanga, Colombia), and the manufacturer’s recommendations for use and dosage were followed.

### 2.4. Production Parameters

On the day of weaning and at the end of the experimental period, all experimental units were individually weighed using a digital scale (Digi-StarW300, Fort Atkinson, WI, USA). Additionally, offered and leftover food was also weighed daily in each of the experimental units. With this recorded information of each experimental unit, daily weight gain (DWG), daily feed intake (DFI), mortality, and Feed/Gain ratio (F:G) were calculated using the formulas described by [1].

### 2.5. Good Practices for Euthanasia and Sample Collection

For sample collection, one animal per experimental unit (a total of 30 pigs) was randomly selected for slaughter on day 30 post-weaning. Euthanasia was performed following the recommendations of the CCAC Guidelines on the Care and Use of Farm Animals in Research [26]. For this, the animals were sedated with a dose of 1 to 2 mg/kg of Azaperone administered intramuscularly and subsequently received inhalation of Nitrox^®^ for two to six minutes per animal. Confirmation of death was performed three minutes after the procedure by detecting the cessation of heartbeats and absence of corneal reflex.

After slaughter, the abdominal region was dissected, the small intestine was removed entirely, and 5 cm samples of the jejunum (middle section of jejunum) were taken. These samples were immediately frozen in liquid nitrogen and stored at −70 °C until analysis at the Animal Biotechnology Laboratory at the headquarters of the Universidad Nacional de Colombia in Medellín. Other 5 cm samples were also obtained from the same segment and stored in 10% buffered formalin for subsequent histotechnical processing Ciro et al. [27]. For the analysis of microbial communities, five grams of luminal contents were sampled from the proximal segment of the ileum, 10 cm anterior to the ileocecal junction. The samples were collected in sterile bags and immediately transferred to plastic vials for deep freezing at −70 °C until processing.

### 2.6. Intestinal Morphometry

Jejunal samples for histotechnical processing were dehydrated in 70%, 80%, 90%, and 100% ethyl alcohol, diaphanized in xylol, and subsequently embedded in paraffin. Moreover, 4 μm thick sections were obtained from each sample using a rotary microtome (Minux^®^ S700 Rotary Microtome, RWD Life Science, Shenzhen, China), stained with hematoxylin and eosin, and placed on microscope slides for morphologic analysis. The slides with histological sections were quantitatively analyzed using digital imaging. For this purpose, a Motican 2300 digital camera (Motic, Hong Kong, China) with a resolution of three megapixels connected to a Leica DLMB optical microscope (Meyer, Houston, TX, USA) was used to capture images of histologic sections at 10× magnification. With the images of each sample, measurements of villus height (VH), villus width, and crypt depth (CD) were taken as described by Barrera et al. [28], using Motic^®^ Images Plus 2.0 image processing software (Motic, Hong Kong, China). Additionally, the variables villus height-to-crypt depth ratio (V:C) and intestinal absorptive area (IAA) were calculated as described by Santos et al. [29]. One slide per experimental unit was used (five animals per treatment). For each slide, measurements were taken from five different fields, with seven measures per field, and these measurements were then averaged.

### 2.7. RNA Extraction and Expression of Enzymatic and Barrier Proteins

RNA was extracted using the UltraClean™ Tissue & Cells RNA Isolation Kit (MO Bio Laboratories Inc Kit, San Diego, CA, USA) following the manufacturer’s instructions. The purity of extracted RNA was confirmed using agarose gel electrophoresis and NanoDrop spectrophotometer (NanoDrop 8000, Thermo Fisher Scientific, Wilmington, DE, USA) at 260 nm wavelength and calculating 260/230 and 260/280 absorbance ratios.

From the previously extracted RNA, cDNA was synthesized using the QuantiTect Reverse Transcription Kit (QIAGEN, Valencia, CA, USA) according to the manufacturer’s recommendations. The resulting cDNA was stored at −20 °C until real-time PCR (RT-PCR) analysis. For RT-PCR, the QuantiNova SYBR Green RT-PCR Kit (QIAGEN, Valencia, CA, USA) was used in a LightCycler 480 RT-PCR System (Roche Diagnostics, Basel, Switzerland). Moreover, 0.5 µg of total cDNA was amplified in 10 µL reaction mix containing 5 µL of QuantiNova 2X SYBR Green RT-PCR Master Mix (QIAGEN, Valencia, CA, USA), 0.1 µL of QuantiNova RT Mix, and 1 µL of reverse and forward primers (Table 2) to a final concentration of 10 µM. For amplification, PCR tubes containing 20 μL of the reaction mixture were subjected to the following thermal cycling profile: 50 °C for 10 min, 95 °C for 2 min, 40 cycles of 95 °C for 2 s, and 60 °C for 10 s, with a final extension at 72 °C for 60 s. Transcripts were quantified using the ΔΔCt method, and the housekeeping gene β-actin was used for normalization.

### 2.8. Metagenomic Sequencing and Bioinformatic Sequence Analysis

The composition of the gut microbial communities of piglets on day 30 post-weaning was evaluated by sequencing the V3–V4 region of the 16S rRNA gene. For this purpose, DNA was extracted and purified from each sample using the FastDNA SPIN Kit according to the manufacturer’s instructions. Amplicons of the V3 and V4 hypervariable regions were then obtained using primers 338F (5′-ACTCCTACGGGAGGCAGCAG-3′) and 806R (5′-GGACTACHVGGGTWTCTAAT-3′) as reported by Niu et al. [33]. Amplicon libraries were generated to be compatible with Illumina paired-end sequencing and subjected to an Illumina Miseq platform (Illumina, San Diego, CA, USA).

The identification and quality control analyses of the sequences in the FASTQ files were performed using the QIIME 2 pipeline (version 2024.07) [34]. Chimeric sequences were identified, extracted, and excluded from the datasets using USEARCH 6.1. Operational taxonomic units (OTUs) with 97% similarity to GenBank sequences were then identified [35]. Finally, the sequences representative of each OTU were selected and taxonomically classified using QIIME 2. During this process, those OTUs with high identity to the sequences reported in GenBank (>97%) were grouped into the same OTU [36].

### 2.9. Statistical Analysis

Expression of gut health-related proteins is shown as the relative expression level of the housekeeping gene used in each qPCR run using the ΔΔCt method. Productive variables, intestinal histology variables, and ΔCt expression levels of each protein were compared between diets using the ANOVA test with an alpha of *p* < 0.05. A Tukey’s test was performed to compare the means between each treatment using the ‘agricolae’ package of the R Studio software (version 4.3.0).

Statistical analysis of the microbial communities was performed using the ‘phyloseq’ package (version 1.42.0) run on the R Studio software [37]. For this purpose, a physeq file was created from the previously obtained databases to perform the diversity and taxonomic composition analyses. Shannon’s diversity index was used to compare the species richness of microbial communities of each diet (alpha diversity), and it was determined using the ‘Microbiome’ package (version 1.24.0) [38]. The comparison between the diets was then made using the ANOVA test [39].

Principal Coordinate Analysis (PCoA) based on Bray–Curtis distances was used to compare the species composition of microbial communities of each diet (beta diversity) using the vegdist function in the ‘vegan’ package (version 2.6-4) [40]. To determine the effect of each diet on the composition of bacterial ecosystems, dissimilarity tests (ANOSIM) and multivariate permutational analysis (PERMANOVA) were performed using the adonis and anosim functions in the ‘vegan’ package [41]. 

Taxonomic analyses were performed using the ‘phyloseq’ package’s taxonomic unit count functions to estimate relative abundance values, and the ggplot2 package (version 3.5.0) was used to plot the results. Correlation maps were generated using the ‘corrplot’ package (version 0.92) [42,43]. Finally, linear discriminant analysis (LDA) effect size (Lefse) analysis was performed using the ‘microbiomeMarker’ package (version 1.8.0) to identify the enriched taxa in each diet using the Kruskal–Wallis rank-sum test (*p* < 0.01 and LDA score > 4.0) [44].

## 3. Results

### 3.1. Production Parameters

During the experimental period, no mortality occurred among any of the animals included in the study. Significant differences (*p* < 0.05) were observed between the diets and all the productive variables evaluated. Final body weight and DWG of the diets containing any of the additives used (D2-AGP, D3-MD, D4-FOS, D5-EO, and D6-SH) were significantly higher than that of D1-BD (*p* < 0.001). D4-FOS was significantly (*p* < 0.001) superior to the other diets regarding DWG and DFI. The best F:G ratio results were obtained in piglets consuming D4-FOS and D5-EO, which had significantly (*p* < 0.001) lower F:G ratio than the rest of the diets evaluated (Table 3).

### 3.2. Intestinal Morphometry

The morphological variables of the jejunum of the piglets at 30 days post-weaning showed differences as a consequence of the diets supplied (Table 4). D4-FOS and D5-EO had higher VH and V:C than the other diets (*p* < 0.01). Regarding IAA, piglets receiving D4-FOS, D5-EO, and D6-SH significantly increased IAA (*p* < 0.001) in relation to D1-BD, D2-AGP, and D3-MD. CD was significantly (*p* < 0.001) higher in D1-BD, D2-AGP, and D3-MD than in the other diets; D4-FOS had the lowest CD with no significant differences with D5-EO. 

**Figure 1:**
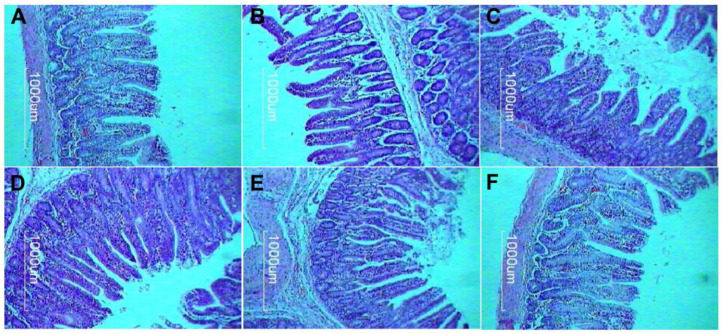
Intestinal morphometric appearances of the jejunum in piglets at 30 days post-weaning. Cross-sections of the jejunum stained with hematoxylin–eosin. Scale bars indicate 1000 μm. (**A**) D1-BD: balanced feed without additives or antibiotics, (**B**) D2-AGP: BD + 150 ppm zinc bacitracin, (**C**) D3-MD: BD + 550 ppm maltodextrin, (**D**) D4-FOS: BD + 300 ppm fructo-oligosaccharides, (**E**) D5-EO: BD + 70 ppm *Lippia origanoides* essential oil, (**F**) D6-SH: BD + 750 ppm sodium humate.

Regarding the morphometric development of jejunal structures in piglets during the first 30 days of the post-weaning period, the D4-FOS and D5-EO diets exhibited the highest degree of morphometric development, with increases in villus length of 20% and 18%, respectively, compared to D1-BD, and 16% and 14%, respectively, in relation to D2-APG. In terms of intestinal absorption area (IAA), which relates to the height and width of villi and crypts, in addition to the D4-FOS and D5-EO diets, D6-SH had 9% more IAA than D2-AGP and was 11% higher when compared to D1-BD (Figure 1).

### 3.3. Barrier Protein Expression

**Figure 2:**
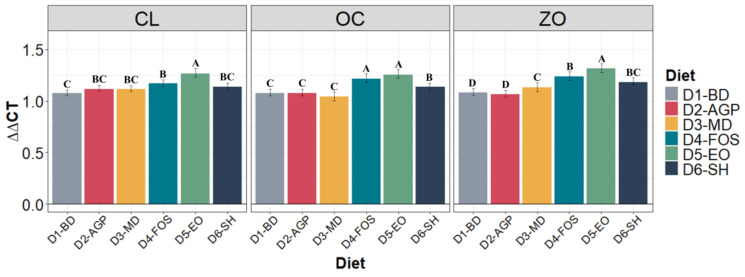
Relative mRNA expression of barrier proteins in the jejunum of piglets supplemented with different types of nutraceutical additives during the 30-day post-weaning period. Differences between diets as determined by Tukey’s test (α ≤ 0.05) are indicated by different letters A, B, C, and D within the same protein. *n* = 5 experimental units per diet. ZO: zonula occludens, CL: claudin, OC: occludin. D1-BD: balanced feed without additives or antibiotics, D2-AGP: BD + 150 ppm zinc bacitracin, D3-MD: BD + 550 ppm maltodextrin, D4-FOS: BD + 300 ppm fructo-oligosaccharides, D5-EO: BD + 70 ppm *Lippia origanoides* essential oil, D6-SH: BD + 750 ppm sodium humate.

Figure 2 describes the results obtained from the gene expression of barrier proteins in the jejunum of piglets 30 days after weaning. No differences (*p* > 0.05) were observed between the diets at day 0 of the experiment for any of the intestinal barrier proteins assessed. Animals fed D5-EO had significantly (*p* < 0.05) higher relative expression of ZO and OC than all other diets in the study. Regarding gene expression of OC, animals receiving D4-FOS and D5-EO showed the highest (*p* < 0.05) relative expression levels. 

### 3.4. Expression of Enzymatic Proteins

**Figure 3:**
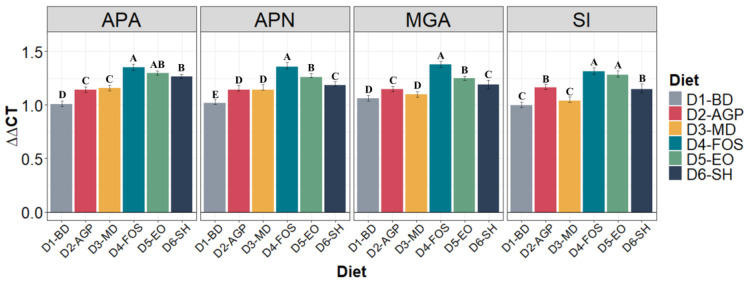
Relative mRNA expression of enzymatic proteins in the jejunum of piglets supplemented with different types of nutraceutical additives during the 30-day post-weaning period. Differences between diets as determined by Tukey’s test (α ≤ 0.05) are indicated by different letters, A, B, C, D, and E, within the same protein. *n* = 5 experimental units per diet. APA: Aminopeptidase A, APN: Aminopeptidase N, MGA: maltase–glucoamylase, SI: sucrase–isomaltase. D1-BD: balanced feed without additives or antibiotics, D2-AGP: BD + 150 ppm zinc bacitracin, D3-MD: BD + 550 ppm maltodextrin, D4-FOS: BD + 300 ppm fructo-oligosaccharides, D5-EO: BD + 70 ppm *Lippia origanoides* essential oil, D6-SH: BD + 750 ppm sodium humate.

The relative expression levels of enzymatic proteins in the jejunum did not show significant differences (*p* > 0.05) between the diets on the first day of the post-weaning period (day 0). At 30 days post-weaning, it was evident that piglets receiving D4-FOS and D5-EO had a higher expression of SI (*p* < 0.05). APA, APN, and MGA had the highest relative expression levels in animals fed D4-FOS, being statistically superior (*p* < 0.05) to all other diets (Figure 3).

### 3.5. Gut Microbial Communities

#### 3.5.1. Diversity Analysis of Gut Microbial Communities

**Figure 4:**
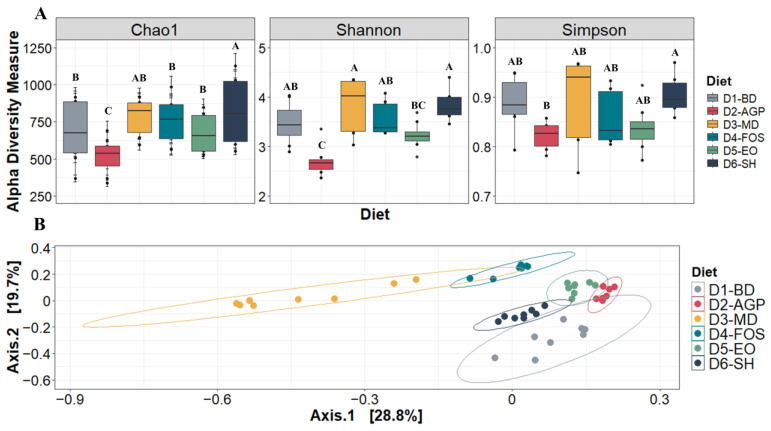
Alpha (A) and Beta (B) diversity of ileal microbial communities of pigs 30 days post-weaning. Part (**A**) shows box and whisker plots of the alpha diversity indices: Chao1, Shannon, and Simpson. Differences between diets as determined by Tukey’s test (α ≤ 0.05) are indicated by different letters: A, B, and C. Part (**B**) presents the Principal Coordinate Analysis (PcoA) based on Bray–Curtis distances showing the distribution of microbial composition of each group and distribution ellipses indicating 95% confidence intervals for each group of piglets fed different diets at 30 days post-weaning. D1-BD: balanced feed without additives, D2-AGP: BD + 150 ppm zinc bacitracin, D3-MD: BD + 550 ppm maltodextrin, D4-FOS: BD + 300 ppm fructo-oligosaccharides, D5-EO: BD + 70 ppm *Lippia origanoides* essential oil, D6-SH: BD + 750 ppm sodium humate.

Alpha diversity was measured using Chao1, Shannon, and Simpson indices. According to these indices, the animals receiving D6-SH and D3-MD at 30 days post-weaning had the highest (*p* < 0.05) levels of diversity. In contrast, D2-AGP had the poorest diversity and was statistically (*p* < 0.05) lower than all other diets regarding Chao1 and Shannon indices (Figure 4A). The PCoA with Bray–Curtis distances in Figure 4B shows that D3-MD, D4-FOS, D5-EO, and D6-SH (which contain non-antibiotic feed additives) are considerably distant from D1-BD. D2-AGP and D5-EO showed the greatest similarity in the composition of all the diets evaluated. The ANOSIM test showed that the diets generated a statistically significant dissimilarity (*p* < 0.001), and it agrees with the PERMANOVA analysis, which found a significant effect (*p* = 0.001) of diets on microbiota composition with an R^2^ = 0.791.

#### 3.5.2. Core Microbiota 

**Figure 5:**
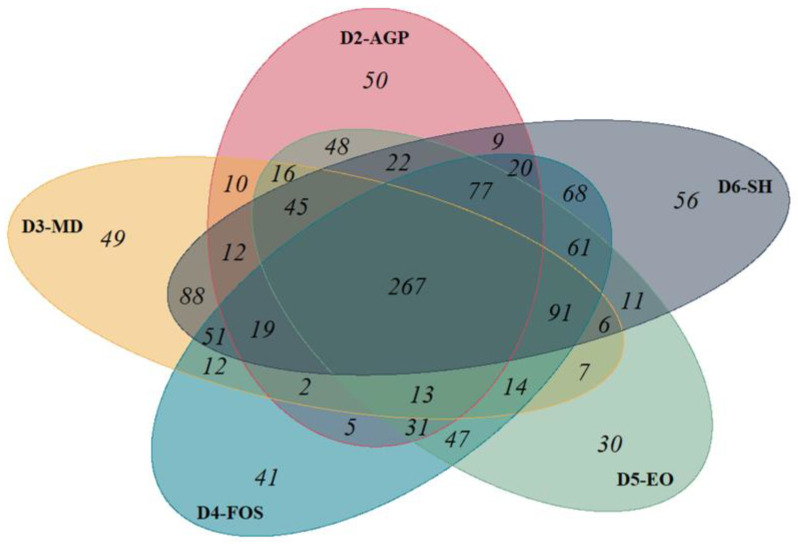
Venn diagram showing the unique and shared taxa in the microbial communities of piglets receiving different feed additives at 30 days post-weaning. The Venn diagram illustrates the distribution and overlap of microbial taxa across the dietary treatments employed in this study. Each colored ellipse represents a distinct diet. The peripheral regions of each ellipse indicate taxa exclusively associated with that particular diet. The intersections between ellipses quantify shared taxa between two or more diets, demonstrating the degree of microbial overlap among treatments. The central region, where all ellipses converge, represents the core microbiome—comprising taxa consistently present across all diets. D2-AGP (red ellipse): BD + 150 ppm zinc bacitracin, D3-MD (yellow ellipse): BD + 550 ppm maltodextrin, D4-FOS (blue ellipse): BD + 300 ppm fructo-oligosaccharides, D5-EO (green ellipse): BD + 70 ppm *Lippia origanoides* essential oil, D6-SH (gray ellipse): BD + 750 ppm sodium humate.

To define the common core microbiota present in the microbial community of piglets receiving diets supplemented with different additives, a group of taxa was identified. This group is represented as the core of the overlapping portions of the circles in the Venn diagram (Figure 5). The core microbiota consists of 267 common taxa present in all the diets. Among the different diets, D6-SH had the highest abundance with 56 unique taxa, while D5-EO had the lowest abundance with only 30 unique taxa.

#### 3.5.3. Taxonomic Analysis

**Figure 6:**
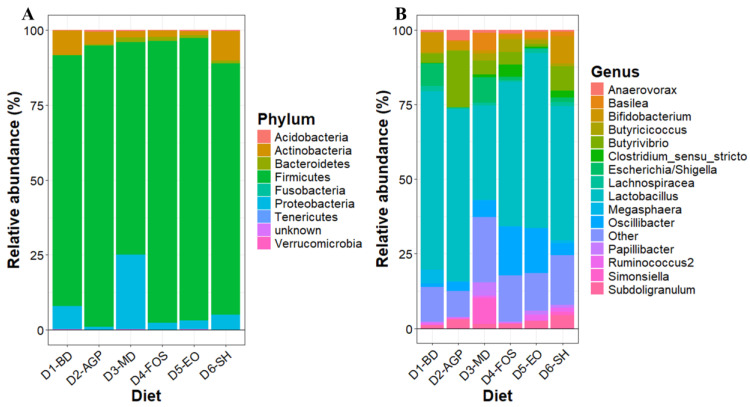
Relative abundance of phyla (**A**) and relative abundance of the top 15 most abundant genera (**B**) in the ileal microbial communities of piglets at 30 days post-weaning. D1-BD: Balanced feed without additives or antibiotics, D2-AGP: BD + 150 ppm zinc bacitracin, D3-MD: BD + 550 ppm maltodextrin, D4-FOS: BD + 300 ppm fructo-oligosaccharides, D5-EO: BD + 70 ppm *Lippia origanoides* essential oil, D6-SH: BD + 750 ppm sodium humate.

Figure 6A shows the relative abundance of bacterial phyla making up the ileal microbial communities of piglets at 30 days post-weaning, considering the different diets fed. The phyla Firmicutes (83.5%), Bacteroidetes (4.7%), Actinobacteria (4.7%), and Proteobacteria (4.2%) represented 97.1% of the microbial communities, and Firmicutes was the dominant phylum in all samples. While D1-BD and D6-SH had the highest relative abundance of Actinobacteria, D1-BD and D3-MD had the highest relative abundance of Proteobacteria. Figure 6B shows the analysis of the 15 most abundant genera in the gut microbial communities of piglets fed different diets. *Lactobacillus* was the most abundant genus in all diet groups; however, the second most abundant genus varied depending on the diet provided. The relative abundance of *Oscillibacter* was higher in D4-FOS and D5-EO, whereas the relative abundance of *Butyrivibrio* was higher in D2-AGP. D6-SH showed high levels of *Bifidobacterium*, the second most abundant genus in this diet, and finally, D1-BD and D3-MD showed *Escherichia/Shigella* as the second most abundant genus in these diets.

#### 3.5.4. Taxa Associated with Diets and Productive Health

**Figure 7:**
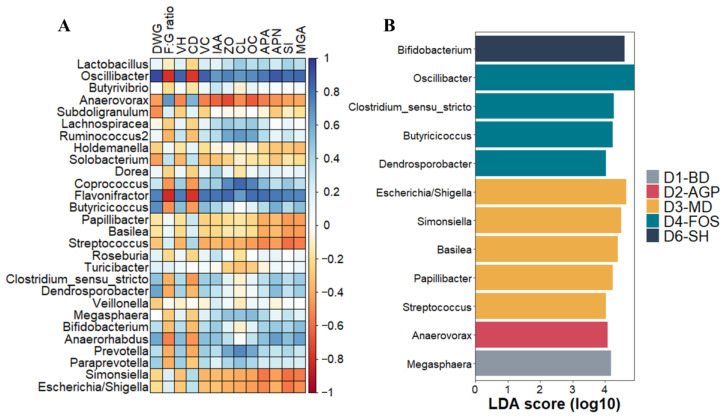
Heat map of Spearman’s correlations between relevant taxa with productive variables and expression of barrier and enzymatic proteins (**A**). LEfse analysis for identifying characteristic taxa in ileal microbial communities (**B**) of piglets 30 days post-weaning with different nutraceutical additives in their diets. The histogram shows the LDA scores of taxa whose abundance differed significantly between diets in the Kruskal–Wallis rank sum test (*p* < 0.01) and which had an LDA score > 4.0 between diets. DWG: daily weight gain, F:G ratio: feed conversion ratio, VH: villus height, CD: crypt depth, V:C: villus height-to-crypt depth ratio, IAA: intestinal absorptive area, APA: Aminopeptidase A, APN: Aminopeptidase N, MGA: maltase–glucoamylase, SI: sucrase–isomaltase, ZO: zonula occludens, CL: claudin, OC: occludin. D1-BD: balanced feed without additives or antibiotics, D2-AGP: BD + 150 ppm zinc bacitracin, D3-MD: BD + 550 ppm maltodextrin, D4-FOS: BD + 300 ppm fructo-oligosaccharides, D6-SH: BD + 750 ppm sodium humate.

A Spearman’s correlation matrix was generated (Figure 7A) to evaluate the relationship between the main taxa at the genus level found in the gut microbial community of piglets at day 30 post-weaning and the different variables evaluated in the study. With this correlation matrix, it was possible to observe a positive association between the productivity variables and the presence of the genera *Oscillibacter*, *Flavonifractor*, and *Butyricicoccus*. In contrast, relative abundances of *Escherichia/Shigella*, *Anaerovorx*, *Subdolingranulum*, and *Solobacterium* were negatively correlated with productivity variables. The relationship between the abundance of microbial taxa and the expression of intestinal proteins examined in this study indicated that both enzymatic and barrier protein overexpression was positively correlated with *Oscillibacter* and *Flavonifractor* taxa. *Coprococcus* and *Ruminococcus2* were closely associated with increased expression of barrier proteins. The increase in *Escherichia/Shigella*, *Streptococcus*, and *Anaerovorx* was associated with decreased relative expression of all proteins tested.

Figure 7B shows the LDA and LEfse analyses identifying the taxa enriched in each diet at the genus level, and it was observed that *Megasphaera* was characteristic of piglets receiving D1-BD. D4-FOS showed the highest number of enriched taxa, including the genera *Oscillibacter spp*, *Clostridium sensu estricto*, *Butyricicoccus*, and *Dendrosporobacter*. In contrast, the microbiota of piglets receiving D5-EO did not exhibit characteristic taxa.

## 4. Discussion

In the present study, it was observed that pigs consuming diets containing FOS (D4-FOS) and *Lippia origanoides* EO (D5-EO) had better productive indices than animals receiving D1-BD and D2-AGP. Concerning the increase in productivity observed in animals receiving EO of *Lippia origanoides*, Dieguez et al. [45] reported that pigs exposed to a 75 ppm dose of EO of *Lippia origanoides*, similar to that used in the present study, had higher weight gains at day 72 than control piglets. 

The results obtained are also in agreement with the results of Patiño et al. [14] and Maya et al. [46], who reported that the use of EO of *Lippia origanoides* in piglets during the post-weaning period improved DWGs between 60 and 86 g/day compared to animals receiving AGPs. The increase in weight gain and feed efficiency in animals supplemented with essential oils has been linked to two main mechanisms: (a) stimulation of digestive enzyme secretion, which increases nutrient digestion/absorption, and (b) stabilization of the intestinal microbial ecosystem, which leads to a lower incidence of digestive disorders such as diarrhea [21].

In addition, improvements in pig productivity associated with the addition of D4-FOS to the diet have previously been linked to the ability of FOS to increase SCFA production due to its fermentation in the gastrointestinal tract by beneficial bacteria. SCFAs play several roles, including modulating the intestinal environment, acidifying the pH, and controlling the growth of potentially pathogenic bacteria [10]. In addition, SCFAs can increase the expression of host defense peptides and regulate the secretion of pro- and anti-inflammatory cytokines to suppress the NF-κB signaling pathway, resulting in improved intestinal immune function [47]. In the present study, piglets supplemented with FOS (D4-FOS) had a DWG of 73 g and a F:G ratio 0.076 points lower than the AGP group (D2-AGP) at 30 days post-weaning; this result is in agreement with the results of the study by Liu et al. [48], using piglets challenged with enterotoxigenic *E. coli* strains during post-weaning.

The present study assessed the relative expression of genes associated with gut health, such as proteins with enzymatic activity (APA, APN, SI, MGA) and proteins that make up the CL, OC, and ZO tight junctions. It was observed that the animals receiving the diet with *Lippia origanoides* essential oil (D5-EO) had the highest relative expression levels of the three barrier proteins studied (OC, CL, and ZO). This beneficial effect of EO of *Lippia origanoides* on the increased expression of intestinal barrier proteins was previously reported by Herrera et al. [1]. These authors observed that 150 ppm of *Lippia origanoides* EO (a higher concentration than the one used in our study) increased the expression (mRNA) of the tight junction proteins CLAU-1, CLAU-4, OCLN, and ZO-1. This result was associated with improved barrier function and intestinal permeability in piglets during the post-weaning period.

The improvement in tight junction protein expression and overall intestinal barrier capacity by essential oils has been associated with the developmental state of intestinal histological structures. EOs act by protecting the intestine from villous atrophy and epithelial cell necrosis, which is related to improved intestinal barrier capacity [49]. Studies conducted in chickens have shown that the relationship between EOs and the intestinal barrier may be directly related to the antioxidant capacity of these compounds. By neutralizing free radicals produced during disease states or stress periods, they contribute to maintaining tissue health and, consequently, the function of tight junctions between enterocytes [50]. This oxidative protection effect of EOs was also reported by Wei et al. [51], who, using an oxidation model in rat jejunum, demonstrated that EO reduced the accumulation of oxygen free radicals in intestinal tissue. This, in turn, resulted in the reduced expression of proinflammatory cytokines such as TNF-α and IL-6 and an increase in occludin expression in the jejunal mucosa, suggesting a protective effect of EOs against oxidative stress and subsequent inflammation and loss of intestinal barrier function [51]. In this regard, it is necessary to develop new studies that delve into the underlying mechanisms of *Lippia origanoides* EO on the intestinal barrier of piglets during the post-weaning period.

Regarding gene expression of enzymatic proteins, in agreement with our findings on the use of EO (D5-EO) and SH (D6-SH) increasing the relative expression of this group of enzymatic proteins, Dieguez et al. [45] found an increase in the enzymatic activity of maltase and sucrase in the jejunum of piglets supplemented with an additive based on a mixture of 75 ppm EO of *Lippia origanoides* and 550 ppm SH (D6-SH). They saw that increased enzyme activity was accompanied by improved development in intestinal morphometry, possibly associated with lower rates of enterocyte loss and turnover, as mature enterocytes may provide greater brush border enzyme activity.

Regarding the effect of the use of FOS (D4-FOS) on the expression of enzymatic proteins in the small intestine of post-weaning piglets, which was the nutraceutical additive that increased the highest expression of these proteins in our study, Liu et al. [48], reported that FOS supplementation at 2.5 g/kg improved maltase and lactase enzyme activity in the duodenum of piglets exposed to enterotoxigenic *E. coli* strains. These authors suggest that this finding may be related to the prebiotic capacity of FOS, as they observed that FOS reduced the adhesion of enteric pathogens to the intestinal epithelium, resulting in a reduced occurrence of intestinal mucosal lesions [48]. In this sense, improvements in gut mucosal health induced by nutraceutical additives may be closely related to increased expression of these proteins in the intestine. According to Wang et al. [52], the reduction in jejunal villus height is associated with decreased brush border enzyme activity, as the production of these enzymes is dependent on enterocyte differentiation and maturation.

Assessing the diversity of gut microbial communities has become critical to understanding the state of the gut microbiota in post-weaning piglets. High levels of alpha diversity have been associated with improved gut health status because it results in “functional redundancy,” meaning that multiple microbial species play similar roles within the microbial community, contributing to a more resilient, resistant, and stable gut ecosystem in the face of both endogenous and exogenous environmental and health challenges faced by piglets [53].

In this study, when alpha diversity was evaluated using Shannon, Chao1, and Simpson indices, it was observed that all groups receiving non-antibiotic food additives (D3-MD, D4-FOS, D5-EO, and D6-SH) had higher diversity than the group receiving AGP (D2-AGP). Specifically, superior diversity was observed in piglets fed MD (D3-MD) and SH (D6-SH). Previous research has studied the effect of fermentable compounds on gut microbial diversity and discovered that adding fermentable oligosaccharides to animal diets can increase the alpha diversity of microbial communities in different gut segments [54]. Yan et al. [55] reported that piglets consuming a dose of 0.4% short-chain FOS in an oxidized oil diet for 28 days post-weaning produced an increase in species richness as assessed by Chao1 and ACE indices compared to animals in the control group. However, other studies have not observed changes in microbial diversity indices, even though FOS consumption altered the balance of microbial species and improved several gut health variables [47,56]. 

These findings suggest variability in the response of microbiota diversity to the inclusion of fermentable oligosaccharides in diets and highlight the importance of considering multiple factors in their effect on gut microbiota composition and diversity. In the present study, microbial communities were evaluated at the ileal level. We suggest the importance of conducting new studies to deepen the understanding of the effect of these types of oligosaccharides in other intestinal segments with longer bacterial fermentation times and their relationship with the intestinal health of piglets during the post-weaning period.

Regarding the effect of using maltodextrin (D3-MD) in piglet diets during the post-weaning period, our study is the first research conducted in pigs to evaluate the effect of MD in piglets. Studies in humans included in a meta-analysis suggest that MD can alter gut microbial communities [57]. Despite these findings presenting contrasting outcomes, it is crucial to further study the effect of this type of carbohydrate and the impact of its dosage in piglet feeding. This is especially important considering its common use as a vehicle for other compounds and additives widely used in pig nutrition. These factors highlight the need for additional research in this area [57]. 

At the taxonomic level, it was observed in the present study that regardless of the diet provided, the microbial communities were mainly composed of Firmicutes, Proteobacteria, Actinobacteria, and Bacteroidetes. The predominant microbial phylum in all piglets at 30 days post-weaning was Firmicutes, representing more than 83% of the communities, followed by Bacteroidetes and Actinobacteria, both phyla with a proportion close to 4.7% of the microbial communities. However, the diets provided had a significant effect on the abundance of different microbial taxa.

The diet containing AGP (D2-AGP) showed a microbiota particularly rich in *Lactobacillus* and a notable presence of *Butyrivibrio* and *Anaerovorax*. Previous studies have reported dietary AGPs’ effects and indicated that they can produce variable results depending on the type and dose used. In this regard, a reduction in the genus *Lactobacillus* has been reported in some cases; in contrast, an increase in the relative abundance of genera such as *Lactobacillus*, *Eggerthella*, *Acetanaerobacterium*, and *Sporacetigenium* has been reported in piglets fed diets supplemented with tylosin [58,59].

Including FOS (D4-FOS) in the piglet diet during the first 30 days after weaning sparked the growth of the genera *Oscillibacter*, *Butyricicoccus*, and *Clostridium sensu stricto*. In a previous study by Zhao et al. [60], it was observed that the use of FOS for 28 days in weaned piglets increased the relative abundance of Bacteroidetes. In addition, there was an increase in the genera *Lactobacillus*, *Bifidobacterium*, and *Prevotella* compared to control piglets. Although this observation is consistent with the increase in *Prevotella* found in our study, no significant increase in *Bifidobacterium* was found in piglets receiving FOS (D4-FOS) [60], a genus that was particularly enriched by the SH (D6-SH) [60].

In this regard, Zhang et al. [47] reported that post-weaning FOS supplementation increased the relative abundance of microbial groups such as *Bacillus* and *Sharpea* in the ileum. These microbial groups have been linked to the ability to ferment complex dietary fibers and, thus, to produce SCFAs and lactic acid in the gut. As reported by these authors, there was a significant increase in the production of these compounds in the ileal contents of piglets fed the FOS diet. FOS, such as those used in this study, are known to be compounds with prebiotic activity because they are carbohydrates that cannot be digested in the upper gastrointestinal tract of piglets due to the β-conformational glycosidic bonds in their molecules. This characteristic makes them susceptible to hydrolysis by intestinal bacteria that possess enzymes such as fructan β-fructosidase or exoinulinase. When fermented by these bacteria, FOS produces several metabolites, most notably butyrate [61].

Finally, the use of AGP (D2-AGP), FOS (D4-FOS), EO (D5-EO), and SH (D6-SH) resulted in a reduction of the genus *Escherichia/Shigella* compared to the groups fed DB (D1-DB) and D3 (D3-MD). This reduction in *Escherichia/Shigella* abundance was particularly pronounced in animals receiving the EO-based additive (D5-EO). This result is consistent with that reported by Mo et al. [62], who found that the use of oils at a concentration of 100 ppm in the diet of piglets had a significant effect on the reduction of potentially pathogenic bacteria such as *Escherichia/Shigella*, *Campylobacter*, *Turicibacter*, *Streptococcus*, and *Treponema*, while there was an increase in potentially beneficial genera such as *Faecalibacterium*, *Alloprevotella*, and *Prevotella* [62].

Spearman’s correlation matrix showed that *Oscillibacter*, *Butyricicoccus*, and *Flavonifractor* were strongly associated with high piglet productivity at 30 days post-weaning. Previous studies have reported a strong relationship between improved production efficiency of pigs and taxa abundance, as well as a greater ability to ferment complex carbohydrates and, therefore, higher metabolic efficiency. Genera such as *Prevotella*, *Butyrivibrio,* and several members of the order *Clostridiales,* including *Oscillibacter*, are some of the enriched taxa identified in the gut microbiota of pigs with higher feed efficiency [63].

The genus *Oscillibacter* is an abundant taxon in the microbiota of pigs, and it is even reported as the second most abundant genus after *Lactobacillus* in some studies [64], a finding that aligns with what our study found in microbial communities of the piglets fed D4-FOS and D5-EO. Regarding its possible biological function, studies have shown that *Oscillibacter* is a valerate-producing bacterium with great probiotic potential due to its ability to produce anti-inflammatory metabolites that modulate Th17 polarization and promote the differentiation of anti-inflammatory Treg/Tr1 cells in the intestine [65,66].

Consistent with our current findings, Xu et al. [67] reported that piglets challenged with enterotoxigenic *Escherichia coli* during weaning showed a significant decrease in the presence of *Flavonifractor* compared to unchallenged piglets. This result suggests that this taxon is often found in piglets with optimal gut health [67]. In addition, it has been reported that weaned piglets from sows supplemented with resveratrol showed an increase in bacteria associated with butyrate production, particularly *Oscillibacter* and *Flavonifractor*, the latter with the ability to metabolize polyphenols for SCFA production [68,69,70]. 

Finally, it should be noted that the results of the LDA and LEfse analyses, used to evaluate the taxa enriched at the genus level in each of the diets, revealed that piglets fed D4 (D4-FOS) presented gut microbial communities particularly enriched in two of the three taxa closely associated with intestinal productivity and health: *Oscillibacter* and *Butyricicoccus*. In this sense, FOS, a compound widely considered a prebiotic, has previously been reported to stimulate the growth of SCFA-producing bacteria such as *Butyricicoccus* [47,71]. 

## 5. Conclusions

The use of different nutraceutical compounds evaluated in this study produced similar and even superior productive results compared to the use of zinc bacitracin (150 ppm) as an AGP during the 30-day post-weaning period. According to the results obtained in the present study, adding FOS or *Lippia origanoides* EO to the diets of post-weaning piglets promoted the best productive results and stimulated the highest expression of enzymatic and barrier proteins in the jejunum. Therefore, FOS (D4-FOS) and EO (D5-EO) are presented as viable alternatives to stimulate gut health and productivity as a replacement for zinc bacitracin as an AGP during the post-weaning period in piglets. At the microbial level, the food additives used in this study differentially modulated the intestinal microbial populations, which could be observed when compared to D1-BD. Concerning zinc bacitracin as AGP (D2-AGP), the use of EO (D5-EO) produced a microbiota of greater similarity compared to the use of zinc bacitracin, and of particular note is the control of potentially pathogenic genera such as *Escherichia/Shigella*. Similarly, the microbial communities of the two fermentable carbohydrate-based feed additives, FOS (D4-FOS) and MD (D3-MD), were more similar to each other, and the ability of FOS to increase the populations associated with SCFA (butyrate) production is noteworthy. It is, therefore, necessary to further explore the relationship between the different microbial balances at the gut level and improvements in piglet productivity. 

## Figures and Tables

**Table 1:** Formula of the basal diet used during the experimental period.

Ingredient	Inclusion Rate (%)
Corn	47.66
Sugar	2.00
Soy Protein Concentrate	0.48
Soybean Meal	20.71
Spray-Dried Animal Plasma	5.00
Fish Meal	3.50
Whey Permeate	8.59
Sweet Whey	4.50
Soybean Oil	3.16
Calcium Carbonate	0.30
Monodicalcium Phosphate	1.34
Salt	0.20
Mineral–vitamin Premix	2.56
Analyzed nutrient composition	
Nutrient	Value
Moisture (%)	10.90
Crude protein (%)	21.89
Fat (%)	5.64
Crude fiber (%)	1.99
Nitrogen-Free Extract (%)	54.39
Metabolizable Energy (Kcal/Kg)	3430
Net Energy (Kcal/Kg)	2568
Ash (%)	5.96
Available Phosphorus (%)	0.55
Calcium (%)	1.07
Lysine (%)	1.45
Methionine (%)	0.41
Met + Cist (%)	0.81
Threonine (%)	0.97
Tryptophan	0.28

**Table 2:** Real-time PCR primers in the analysis of the relative expression of gut health-related proteins.

Gen	Sequence		Annealing Temperature °C	GenBank/Reference
Claudin-1 (CL)	Forward	5′-GCCACAGCAAGGTATGGTAAC-3′	60	[30]
	Reverse	5′-AGTAGGGCACCTCCCAGAAG-3′		
Occludin (OC)	Forward	5′-GGAGGAAGACTGGATCAGGGA-3′	62	[30]
	Reverse	5′-AGCAGCAGCCATGTACTCTT-3′		
Zonula occludens-1 (ZO	Forward	5′-TGGCATTATTCGCCTTCATAC-3′	59	[30]
	Reverse	5′-AGCCTCATTCGCATTGTTT-3′		
Maltase–glucoamylase (MGA)	Forward	5′-CCAGAGCTTGTCACTCAGCA-3′	56	[31]
	Reverse	5′-GCACGTCATAGGGGATCTGG-3′		
Sucrase–isomaltase (SI)	Forward	5′-TGGCCATCCAGTCATGCC-3′	56	[31]
	Reverse	5′-CCACCACTCTGCTGTGGA-3′		
Aminopeptidase A (APA)	Forward	5′-CCTCCGGCGTCTGTGTTA-3′	56	[31]
	Reverse	5′-TGGATTCAGCTCACAGCT-3′		
Aminopeptidase N (APN)	Forward	5′-ACATCACTCTCATCCACCCT-3′	58	[31,32]
	Reverse	5′-GCAATCACAGTGACAACTCG-3′		
β-actin	Forward	5′-CCAGCACGATGAAGATCAAGA-3′	60	AY550069.1
	Reverse	5′-AATGCAACTAACAGTCCGCCTA-3′		

**Table 3:** Effect of different nutraceutical feed additives on the productive variables of piglets during the 30-day post-weaning period.

Variable	Mean	D1-BD	D2-AGP	D3-MD	D4-FOS	D5-EO	D6-SH	SEM	*p*-Value
Initial Weight	6.61	6.64	6.60	6.64	6.61	6.56	6.57	0.015	0.851
Final Weigh	20.99	19.60 ^E^	20.33 ^D^	20.856 ^C^	22.54 ^A^	21.76 ^B^	20.84 ^C^	0.179	<0.001
DWG (kg)	0.479	0.432 ^E^	0.458 ^D^	0.474 ^C^	0.531 ^A^	0.506 ^B^	0.476 ^C^	0.006	<0.001
DFI (kg)	0.658	0.624 ^D^	0.643 ^CD^	0.652 ^C^	0.706 ^A^	0.677 ^B^	0.646 ^C^	0.005	<0.001
F:G ratio	1.374	1.445 ^D^	1.404 ^C^	1.376 ^BC^	1.329 ^A^	1.338 ^A^	1.358 ^B^	0.007	<0.001

Different superscripts ^A, B, C, D, E^ in the same row indicate differences (Tukey’s test) between diets (α < 0.05). SEM: standard error of the mean. DWG: daily weight gain; Final Weight: body weight at 30 days post-weaning. DFI: daily feed intake; F:G ratio: Feed/Gain ratio. D1-BD: balanced feed without additives or an; D2-AGP: BD + 150 ppm zinc bacitracin; D3-MD: BD + 550 ppm maltodextrin; D4-FOS: BD + 300 ppm fructo-oligosaccharides; D5-EO: BD + 70 ppm *Lippia origanoides* essential oil; D6-SH: BD + 750 ppm sodium humate.

**Table 4:** Effect of different nutraceutical feed additives on morphometric variables of the jejunum in piglets at the end of the 30-day post-weaning period.

Variable	Mean	D1-BD	D2-AGP	D3-MD	D4-FOS	D5-EO	D6-SH	SEM	*p*-value
VH (µm)	586.21	524.73 ^C^	547.46 ^C^	561.36 ^BC^	655.64 ^A^	640.26 ^A^	587.80 ^B^	9.59	<0.001
CD (µm)	116.13	127.23 ^A^	124.71 ^A^	121.35 ^A^	103.72 ^C^	107.07 ^BC^	112.70 ^B^	0.26	<0.001
V:C	5.05	4.12 ^C^	4.39 ^C^	4.63 ^BC^	6.32 ^A^	5.98 ^A^	5.22 ^B^	0.16	<0.001
IAA (mm^2)^	6.02	5.57 ^C^	5.69 ^BC^	5.84 ^B^	6.44 ^A^	6.31 ^A^	6.28 ^A^	0.40	<0.001

Different superscripts ^A, B, C^ in the same row indicate differences (Tukey’s test) between diets (α < 0.05). SEM: standard error of the mean. VH: villus height, CD: crypt depth, V:C: villus height-to-crypt depth ratio, IAA: intestinal absorptive area. D1-BD: balanced feed without additives or antibiotics; D2-AGP: BD + 150 ppm zinc bacitracin; D3-MD: BD + 550 ppm maltodextrin; D4-FOS: BD + 300 ppm fructo-oligosaccharides; D5-EO: BD + 70 ppm *Lippia origanoides* essential oil; D6-SH: BD + 750 ppm sodium humate.

## Data Availability

The datasets generated for this study are available upon request to the corresponding author.
